# Regulatory effects of post-translational modifications on zDHHC *S*-acyltransferases

**DOI:** 10.1074/jbc.REV120.014717

**Published:** 2020-08-17

**Authors:** Filip Zmuda, Luke H. Chamberlain

**Affiliations:** Strathclyde Institute of Pharmacy and Biomedical Sciences, University of Strathclyde, Glasgow, Scotland, United Kingdom

**Keywords:** protein acylation, post-translational modification (PTM), protein palmitoylation, protein chemical modification, S-acylation, zDHHC enzyme, phosphorylation, ubiquitination, acetylation, methylation

## Abstract

The human zDHHC *S*-acyltransferase family comprises 23 enzymes that mediate the *S*-acylation of a multitude of cellular proteins, including channels, receptors, transporters, signaling molecules, scaffolds, and chaperones. This reversible post-transitional modification (PTM) involves the attachment of a fatty acyl chain, usually derived from palmitoyl-CoA, to specific cysteine residues on target proteins, which affects their stability, localization, and function. These outcomes are essential to control many processes, including synaptic transmission and plasticity, cell growth and differentiation, and infectivity of viruses and other pathogens. Given the physiological importance of *S*-acylation, it is unsurprising that perturbations in this process, including mutations in *ZDHHC* genes, have been linked to different neurological pathologies and cancers, and there is growing interest in zDHHC enzymes as novel drug targets. Although zDHHC enzymes control a diverse array of cellular processes and are associated with major disorders, our understanding of these enzymes is surprisingly incomplete, particularly with regard to the regulatory mechanisms controlling these enzymes. However, there is growing evidence highlighting the role of different PTMs in this process. In this review, we discuss how PTMs, including phosphorylation, *S*-acylation, and ubiquitination, affect the stability, localization, and function of zDHHC enzymes and speculate on possible effects of PTMs that have emerged from larger screening studies. Developing a better understanding of the regulatory effects of PTMs on zDHHC enzymes will provide new insight into the intracellular dynamics of *S*-acylation and may also highlight novel approaches to modulate *S*-acylation for clinical gain.

Proteins are essential functional and structural components of living organisms, and an estimated 20,000 protein coding genes have been identified in higher organisms, such as humans (1) and mice (2). However, direct expression of these genes is not sufficient to account for the complexity of life processes, and additional means of extending protein diversity are required. This is achieved through alternative splicing during mRNA maturation and protein post-translational modifications (PTMs). In the case of humans, this allows the body to extend the proteome to an estimated 0.62–6.13 million different protein species that are necessary to sustain life (3).

*S*-Acylation represents one of over 200 different types of PTMs (4, 5), but it has come to prominence recently following several major breakthroughs. One important advance has been the development of chemical biology methodologies that have facilitated MS-based profiling of the *S*-acylated proteome in a range of different cells and organisms (6, 7). This has revealed that over 4,000 human and mouse proteins are *S*-acylated, of which ∼140 mouse and 350 human proteins have, to date, been validated using other techniques (8). These *S*-acylated proteins include soluble signaling proteins, membrane receptors, ion channels, transporters, molecular scaffolds, and chaperones (9). Functional roles of *S*-acylation in all the mammalian physiological systems are continually being uncovered, including important roles in synaptic transmission and plasticity (10, 11), cardiac electrophysiology (12, 13), hormone release and response pathways (11, 14), and immune cell function (15). In addition, links between *S*-acylation and pathological processes are also emerging, including in viral and bacterial infection (16), intellectual disability, epilepsy, schizophrenia, Huntington's disease, diabetes, and cancer (9, 17–20). Indeed, recent studies have shown that *S*-acylation stabilizes the immune checkpoint protein PD-L1, allowing cancer cells to evade the immune system (21). As a result, there is growing interest in *S*-acylation and the enzymes that control this process.

*S*-Acylation involves the attachment of fatty acids to cysteine residues on proteins through a labile thioester linkage (9). The attachment of the palmitate (C16:0) fatty acid, known as palmitoylation, is the most common type of *S*-acylation, although stearate (C18:0), oleate (C18:1), and other longer chain fatty acids can also be utilized to a lesser extent (22–27). The attachment of these fatty acids can affect proteins in a variety of ways. At the simplest level, the hydrophobic acyl chains mediate membrane attachment of a variety of soluble proteins, including Ras, Src, and G_α_ subunits (28). *S*-Acylation is also a recognized intracellular sorting signal that controls the movement of proteins through the secretory and endosomal networks (28). Indeed, a recent study suggested that *S*-acylation plays a central role in anterograde intra-Golgi transport of newly synthesized proteins by mediating enrichment of proteins in the rims of Golgi cisternae from where vesicle budding takes place (29). Targeting to the rims of the cisternae was suggested to reflect an intrinsic affinity of *S*-acylated proteins for curved membranes (29). Lateral segregation is also proposed to operate at other intracellular membranes as *S*-acylation appears to be a signal for protein movement into cholesterol-rich raft membranes (30). Other prominent effects of *S*-acylation include regulating protein-protein interactions or protein stability, the latter effect through interplay with ubiquitin-dependent degradation processes (9).

*S*-Acylation is widespread in eukaryotes, and breakthrough studies in the yeast *Saccharomyces cerevisiae* showed that this process is enzyme-mediated (31, 32). The defining feature of *S*-acyltransferase enzymes is a 51-amino acid zinc-finger cysteine-rich domain (CRD) that contains a catalytic DHHC (apartate-histidine-histidine-cysteine) tetrapeptide (33, 34). Twenty-three of these “zDHHC” enzymes are present in mammalian species (35), and they have been the subject of intense interest since their discovery. All zDHHC enzymes are predicted to contain between four and six membrane-spanning domains, with the catalytic DHHC domain present in a cytoplasmic loop, and in mammalian cells, these enzymes localize in an isoform-specific manner to the endoplasmic reticulum, Golgi, endosomes, and the plasma membrane (36–39). The process of *S*-acylation involves a two-step ping-pong mechanism, where the deprotonated and nucleophilic DHHC cysteine first undergoes autoacylation, followed by transfer of the acyl group to a cysteine residue on a target protein (40, 41).

A recent breakthrough study described the crystal structure of human zDHHC20 and zebrafish zDHHC15 (39, 42). The structure revealed that the transmembrane domains of these enzymes form a tepee-like arrangement that accommodates the acyl chain of the acyl-CoA, with the cysteine of the DHHC motif positioned at the cytosolic face of the membrane at the base the cavity (39, 42). The aspartate residue of the DHHC motif acts to polarize the second histidine, which deprotonates the catalytic cysteine to form a reactive thiolate nucleophile. This cysteine thiolate then attacks the thioester carbonyl carbon of the acyl-CoA molecule to form the autoacylated intermediate (39, 42). The DHHC-CRD also contains two zinc fingers that coordinate two zinc ions (Zn^2+^), which are important to maintain the structural integrity of this domain (39, 42). In addition, important contacts are made between the DHHC-CRD and conserved motifs in the C terminus of zDHHC enzymes (39, 42).

Following autoacylation of zDHHC enzymes, the acyl chain is either hydrolyzed or transferred to a reactive cysteine in a substrate protein (40, 41). Some zDHHC enzymes display a lack of substrate selectivity and modify accessible and reactive cysteines in a diverse array of protein targets (43, 44). In contrast, other zDHHC enzymes are highly selective and recognize specific domains or motifs in their substrate targets as an essential prerequisite to *S*-acylation (9, 45). This combination of selectivity and promiscuity is likely essential to allow the *S*-acylation of a large and diverse set of cellular proteins.

Despite growing knowledge of the substrate targets and physiological roles of the zDHHC enzyme family, the means through which these enzymes are regulated are poorly understood. However, emerging evidence highlights a role for different PTMs in regulating several members of the zDHHC family. This review will discuss established roles of PTMs in regulating the stability, localization, and function of zDHHC enzymes and speculate on the potential role of modifications identified in larger proteomic studies, using available bioinformatics and structural data.

## Post-translational modifications of zDHHC enzymes

**Figure 1.**
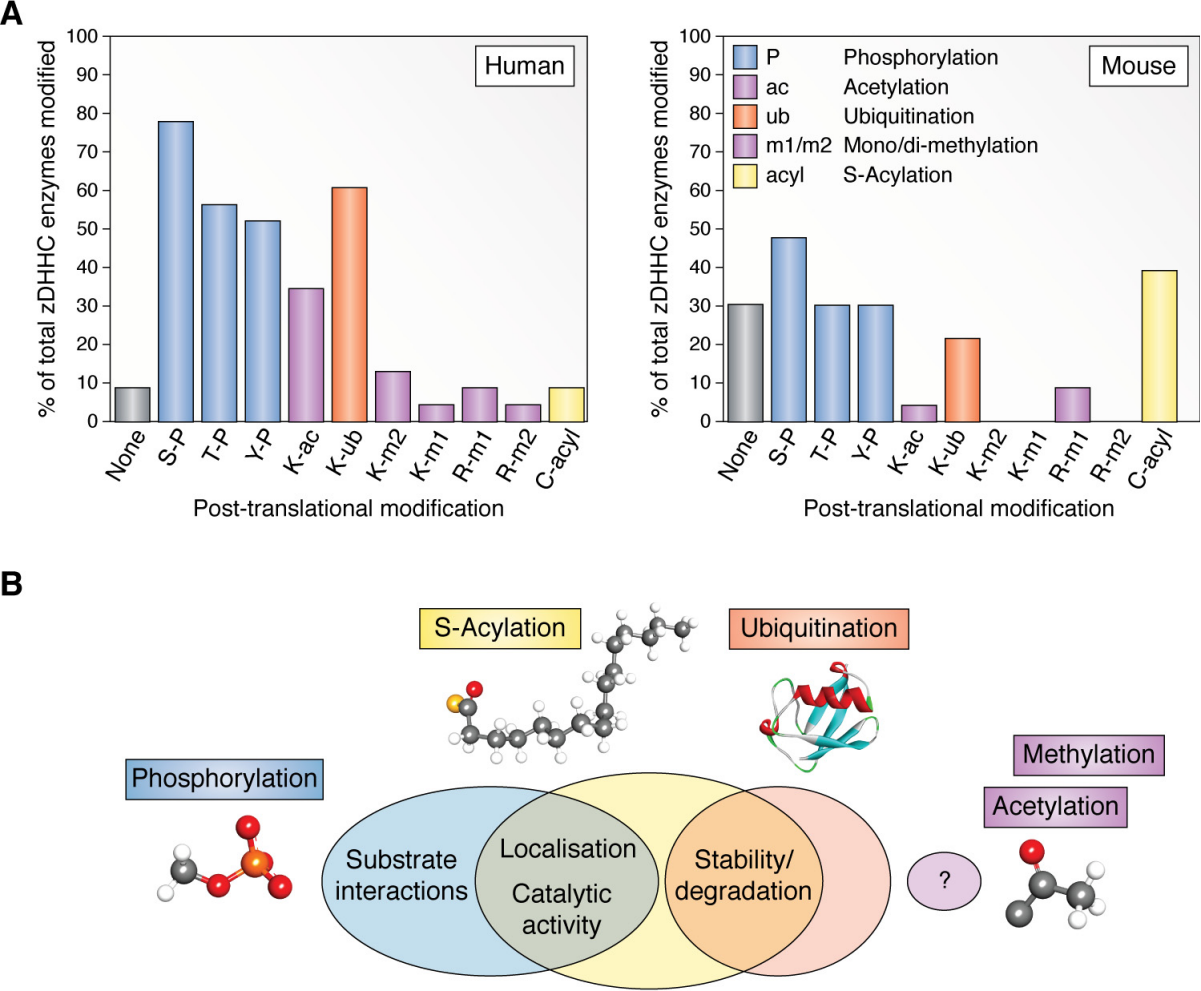
**Post-translational modifications of zDHHC enzymes.** *A*, percentage of the primary isoforms of the 23 zDHHC human and mouse enzymes for which the listed post-translational modifications have been reported. *B*, regulatory effects on zDHHC enzymes of the post-translational modifications discussed in this review. Data were obtained by searching the PhosphoSitePlus (46), dbPTM (47), and SwissPalm (8) online databases. All included modifications are supported by at least one experimental data set. The synthetic ubiquitin structure (1OGW) (48) was obtained from the RCSB Protein Data Bank (49). *P*, phosphorylation; *ac*, acetylation; *ub*, ubiquitination; *m1*, monomethylation; *m2*, dimethylation; *acyl*, acylation.

Traditional labeling and detection methodologies have identified PTMs that regulate many cellular proteins, albeit with a bias toward phosphorylation. Although these traditional approaches have been hugely valuable, they are time-consuming and often focus on detailed analysis of single sites of modification within a single protein of interest. In recent years, the vast increase in the use of -omics technologies to investigate cellular processes and pathways together with prediction algorithms have produced a wealth of information on the different chemical modifications that have been detected or are predicted to occur on cellular proteins. Whereas this has provided a detailed catalogue of PTMs on any single protein, it is important to note that the majority of these lack validation, and the stoichiometry of modification is unclear. The relative abundance of different PTMs in human and mouse zDHHC enzyme isoforms is highlighted in Fig. 1*A*, with a focus on phosphorylation, ubiquitination, *S*-acylation, acetylation, and methylation. Collectively, these types of PTM have been shown to exert a variety of regulatory effects on different proteins, including modulating protein-protein interactions, protein activity, stability, degradation, and subcellular localization (50–56). Specific regulatory effects of phosphorylation, ubiquitination, and *S*-acylation on zDHHC enzymes have been described, whereas mechanistic effects of acetylation and methylation on these enzymes have not yet been reported (Fig. 1*B*).

Globally, phosphorylation represents the most studied PTM, which has also been found to be highly abundant in the human proteome (53). Therefore, it is not surprising that phosphorylations of serine, threonine, and tyrosine residues are prominent modifications of zDHHC enzymes (Fig. 1*A*). Ubiquitination is also detected on many zDHHC enzymes, whereas lysine acetylation and *S*-acylation are prominent in the human and mouse enzyme families, respectively. The latter is likely a consequence of the divergent proteomics screening efforts that have been, to date, conducted in these species. In both human and mouse studies, arginine and lysine methylation of zDHHC enzymes have been reported less commonly (Fig. 1*A*). Of the 23 zDHHC enzymes, current data suggest that zDHHC5 may be the most extensively modified, exhibiting eight different PTM types in humans and five different PTM types in mice. In the human isoform, phosphorylation and ubiquitination are the predominant modifications. However, a caveat when comparing PTMs of different zDHHC enzymes is that detection will be influenced by the relative abundance of these enzymes in the source tissue.

## Regulation of zDHHC enzymes by phosphorylation

**Figure 2.**
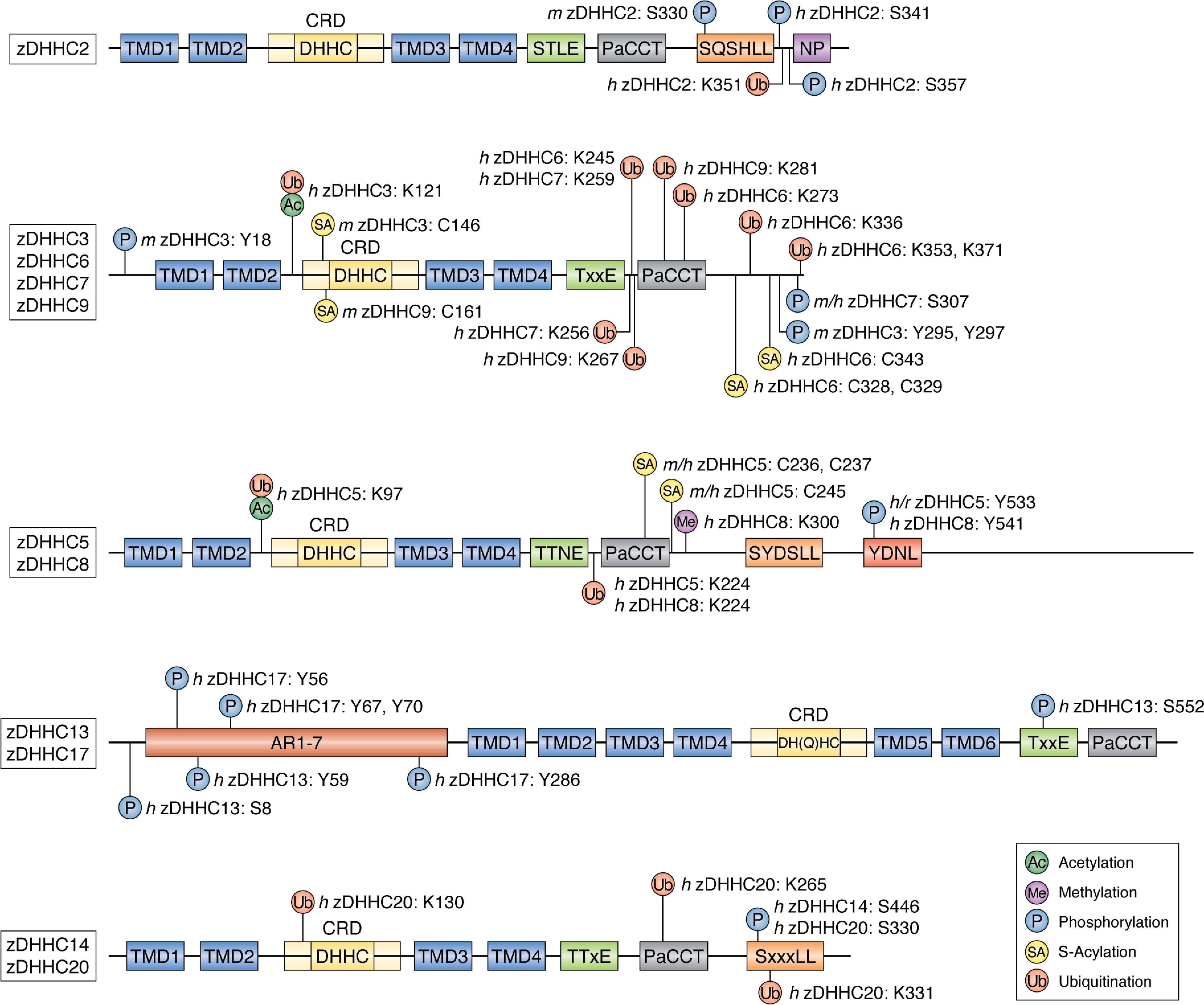
**Diagram representing domains and motifs of selected zDHHC enzymes and key post-translational modifications and the residues modified.** Post-translational modification data were obtained by searching the PhosphoSitePlus (46), dbPTM (47), and SwissPalm (8) online databases. All included modifications are supported by at least one experimental data set. *Ac*, acetylation; *Me*, methylation; *P*, phosphorylation; *Pal*, palmitoylation; *Ub*, ubiquitination; *AR*, ankyrin-repeat domain; *CRD*, cysteine-rich domain; *PaCCT*, palmitoyl conserved C terminus; *h*, human; *m*, mouse; *r,* rat.

Phosphorylation is known to modulate a multitude of protein-protein interactions (53), and recent work showed phospho-dependent regulation of substrate binding by the Golgi enzyme zDHHC13. Specifically, phosphorylation of zDHHC13 at Ser^8^ by ataxia telangiectasia and Rad3-related kinase enhanced its interaction with the substrate protein melanocortin 1 receptor (MC1R) (57, 58). *S*-Acylation of MC1R is associated with enhanced receptor signaling, increased synthesis of melanin, and activation of cell-protective DNA-repair pathways (57). Furthermore, enhancing the *S*-acylation of MC1R variants that are linked to red hair and increased susceptibility to melanoma was shown to prevent melanomagenesis in mice (57). zDHHC13 is closely related to zDHHC17, and these are the only zDHHC isoforms to possess cytosolic N-terminal ankyrin (ANK) repeat domains, which have been shown to play an important role in protein substrate recognition and binding (45). However, Ser^8^ is outside of the ankyrin-repeat domain of zDHHC13 (amino acids 43–277) (Fig. 2), and thus its role in substrate binding is unclear. It will be important to determine the mechanism through which phosphorylation of this amino acid affects MC1R interaction and whether the effect of this PTM extends to other zDHHC13 substrates. For example, is Ser^8^ part of the MC1R-binding site or does phosphorylation of this residue alter the structure or presentation of the ANK domain to facilitate binding?

Interestingly, Tyr^59^ in zDHHC13 is also modified by phosphorylation (59) (Fig. 2). This site is of particular interest as it is in the ankyrin-repeat domain of zDHHC13, and recent crystallographic data showed that the corresponding residue in zDHHC17 (Tyr^67^) is part of the substrate interaction site of this enzyme. Moreover, a tyrosine-to-alanine substitution resulted in a 5-fold drop in the affinity of zDHHC17 for the SNAP25 substrate protein (60). As zDHHC13 also binds to SNAP25, it can be envisaged that the addition of negative phosphate moieties to Tyr^59^ could disrupt substrate interactions of zDHHC13. Phosphoregulation may also affect zDHHC17 substrate interactions as several residues in this Golgi-localized enzyme, including Tyr^56^, Tyr^67^, and Tyr^70^ in the first ANK domain of zDHHC17 and Tyr^286^ in the last ANK domain of this enzyme, have also been found to be phosphorylated in proteomics experiments (59, 61) (Fig. 2).

The Golgi-localized enzyme zDHHC3 (37, 43) has also been suggested to display phospho-dependent regulation of substrate interactions. In mouse neuroblastoma N2A cells, phosphorylation of zDHHC3 Tyr^18^ by fibroblast growth factor (FGF) receptor and Tyr^295^/Tyr^297^ by Src kinase (Fig. 2) was suggested to affect enzyme activity and interaction with the neuronal cell adhesion molecules (NCAM140/NCAM180) following FGF2 stimulation (62). Regulation of zDHHC3 substrate binding is particularly interesting, as previous work suggested that this highly active and promiscuous enzyme does not display detectable binding to at least some of its substrates (43). Therefore, it will be interesting to explore whether the phosphorylation events that impact NCAM140/180 interaction also affect *S*-acylation of other substrates of this enzyme. Interestingly, these phosphorylation sites in zDHHC3 do not appear to be conserved in zDHHC7 (Fig. 2), and therefore unique phosphorylation profiles of these highly similar enzymes offer potential for their differential regulation.

**Figure 3.**
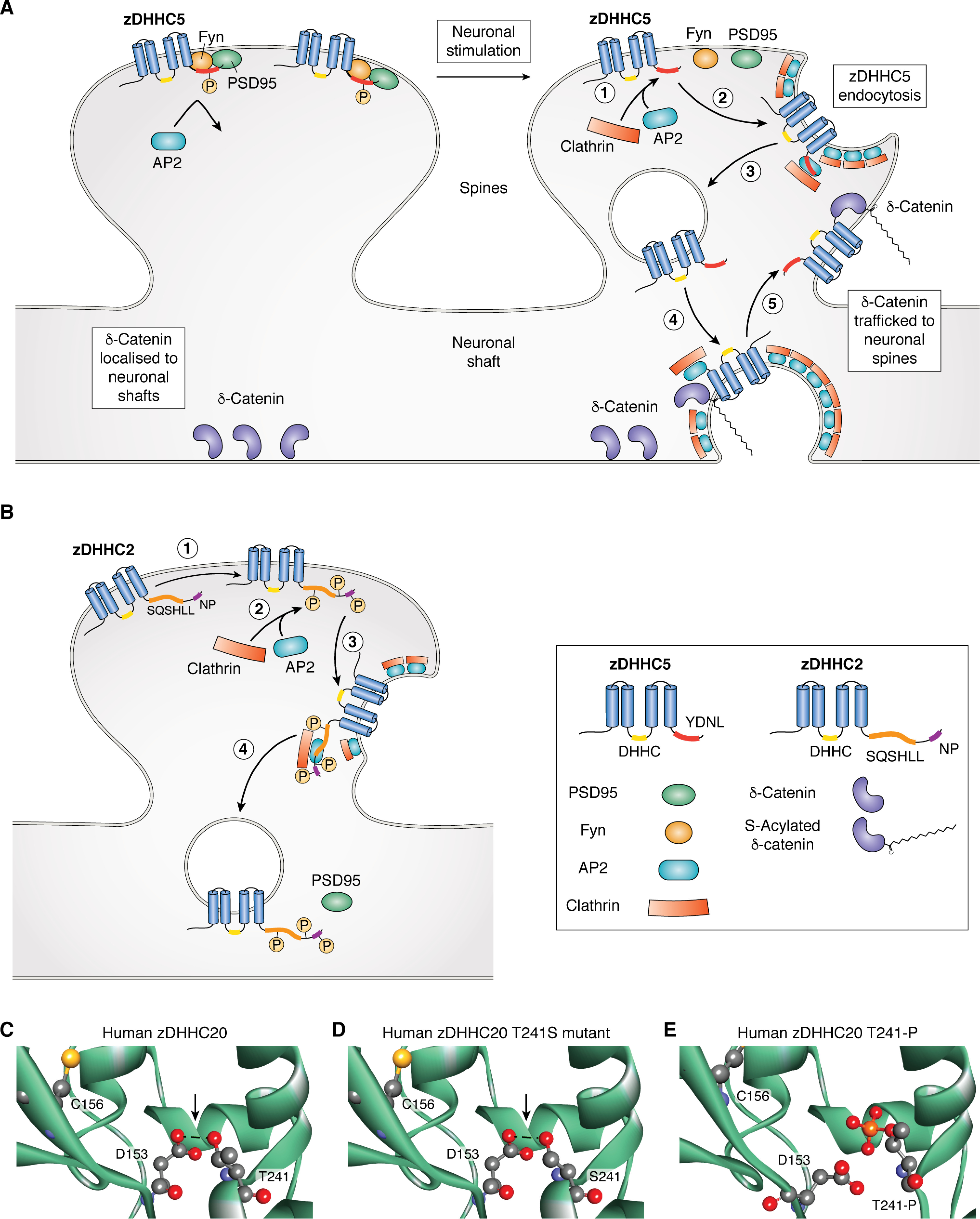
**Phosphoregulation of zDHHC enzymes.** *A*, effects of phosphorylation on zDHHC5 localization and substrate interaction. Under basal conditions, zDHHC5 is localized to neuronal spines in a complex with PSD95 and Fyn kinase, whereas δ-catenin is predominantly localized to neuronal shafts. Fyn kinase phosphorylates zDHHC5 Tyr^533^ in the C-terminal YDNL motif, which prevents binding of the endocytic AP2 adaptor protein. Following neuronal stimulation, Fyn kinase is inactivated, and the zDHHC5-PSD95-Fyn complex dissociates. In the absence of Tyr^533^ phosphorylation, AP2 is able to bind to zDHHC5 (*1*), and the *S*-acyltransferase then undergoes endocytosis (*2*). zDHHC5 is then translocated to neuronal shafts (*3*), where it is able to *S*-acylate δ-catenin (*4*). Both zDHHC5 and modified δ-catenin are then trafficked to neuronal spines (*5*), where the two proteins co-localize. For clarity, *S*-acylation is only shown on δ-catenin (although PSD95, Fyn kinase, and zDHHC5 also undergo *S*-acylation). *B*, effects of phosphorylation on zDHHC2 localization. In its nonmodified form, zDHHC2 is primarily localized to post-synaptic membranes and undergoes minimal endocytotic cycling. However, phosphorylation of specific C-terminal residues between SQSHLL and NP motifs, and the SQSHLL motif serine residue (Ser^330^ in mouse zDHHC2) (*1*) facilitates recruitment of endocytotic adaptor proteins (*2*), which is proceeded by endocytosis (*3* and *4*) of zDHHC2. This trafficking of zDHHC2 allows access to its substrate protein PSD95. *C–E*, modeling effects of phosphorylation in the TT*X*E motif of zDHHC20. *C*, the crystal structure of the human zDHHC20 (6BMM) enzyme (39), which was obtained from the RCSB Protein Data Bank (49), showing the catalytic cysteine (*C156*), and hydrogen bonding (*arrow*) between the DHHC aspartate (*D153*) and the C-terminal TT*X*E threonine (*T241*). *D*, predicted hydrogen bonding (*arrow*) between the DHHC aspartate (*D153*) and the TS*X*E serine (*S241*) in a zDHHC20 T241S mutant. *E*, predicted structure of the zDHHC20 enzyme bearing a phosphothreonine (*T241-P*). The nonstandard CME^156^ residue in the 6BMM structure was point-mutated to Cys^156^ as per the zDHHC20 6BMN structure (39). The post-translational modification was introduced into the zDHHC20 crystal structure using the AmberTools 18.0 software package and the FFPTM forcefield (65). The modified structure underwent energy minimization, and all models were visualized using the Discovery Studio 2020 software.

Phosphorylation has also been suggested to control the trafficking of zDHHC5 and zDHHC2, which are localized to post-Golgi membrane compartments in neurons. These effects of phosphorylation are thought to involve regulated interactions of the zDHHC enzymes with endocytic adaptor proteins. Under basal conditions, zDHHC5 localizes to dendritic spines in rat hippocampal neurons, where it associates with postsynaptic density protein 95 (PSD95) and Fyn kinase (63). Under these conditions, the overlap of zDHHC5 and its substrate δ-catenin is low (63). Following neuronal stimulation, however, zDHHC5 displays a shift in localization, which allows it to access and *S*-acylate δ-catenin, which facilitates movement of this protein to spines where it promotes stabilization of N-cadherin at synapses, synaptic enlargement, and the recruitment of α-amino-3-hydroxy-5-methyl-4-isoxazolepropionic acid glutamate receptors to these sites (64). How does neuronal stimulation lead to zDHHC5 redistribution? This effect appears to be linked to phosphorylation of Tyr^533^, which is present within a ^533^YDNL endocytotic motif (63, 64) (Fig. 2). Under resting conditions, this site is phosphorylated by Fyn kinase and therefore not active (Fig. 3*A*). However, following neuronal stimulation, the zDHHC5-Fyn interaction is reduced, allowing dephosphorylation of Tyr^533^ and subsequent recruitment of the μ subunit of the clathrin adaptor AP2, followed by clathrin-mediated endocytosis of zDHHC5 and co-localization with δ-catenin (63, 64) (Fig. 3*A*). Interestingly, the C-terminal tail of zDHHC5 appears to exhibit extensive serine and threonine phosphorylation (46). This hints toward the possible phosphoregulation of other facets of this enzyme, although the biological relevance of many of these modifications is yet to be established.

zDHHC2 also displays a dynamic localization, and it was shown to cycle between recycling endosomes and the post-synaptic plasma membrane to regulate activity-dependent *S*-acylation of the post-synaptic scaffold PSD95 (36, 66). Interestingly, phosphorylation may also regulate this dynamic cycling of this enzyme by affecting the efficacy of trafficking signals (67) (Fig. 3*B*). Analysis of the localization of mouse zDHHC2 in neuroendocrine PC12 cells and hippocampal neurons identified two potential endocytic signals in the C-terminal tail, which when mutated led to enhanced accumulation at the plasma membrane: (i) a nonconventional dileucine motif (S*XXX*LL, where *X* represents any amino acid) at amino acid positions 330–335, which differs from the conventional acidic (D/E)*XXX*LL motif that interacts with clathrin adaptors and (ii) an Asn-Pro (NP) motif at amino acid positions 357 and 358 (67) (Fig. 2). It was suggested that phosphorylation of Ser^330^ in the S*XXX*LL motif could make this more like a conventional acidic dileucine motif and allow recognition by AP2. Indeed, introduction of a phospho-null mutation (alanine) at Ser^330^ led to increased abundance of zDHHC2 at the plasma membrane, whereas a phosphomimetic mutant (aspartic acid substitution) had no effect (67). Interestingly, additional phosphomimetic mutations introduced in the vicinity of the dileucine or NP motif increased the abundance of zDHHC2 inside the cell, suggesting that phosphorylation of specific sites in the C terminus may enhance recognition of these endocytic motifs by AP2, perhaps by inducing local conformational changes that unmask the endocytic sites (67). Specifically, phosphomimetic, but not phospho-null, substitutions at Ser^356^ and Thr^361^ led to an increased endosomal localization of zDHHC2, and this effect required an intact NP motif. This suggests that phosphorylation of Ser^356^ and Thr^361^ might enhance the efficacy of the NP motif as an endocytic signal. In contrast, the enhanced endosomal localization of zDHHC2 carrying phosphomimetic substitutions of Ser^344^, Ser^345^, Thr^340^ and Thr^342^ depended on an intact dileucine motif, suggesting that phosphorylation of these residues enhances this endocytic signal (Fig. 3*B*). The key serine of the S*XXX*LL motif (Ser^330^) has been reported as a phosphorylation site in a single phosphoproteome study of mouse pancreatic cells, whereas modification of Ser^341^ and Ser^357^ in human zDHHC2, which are present at the same position as Thr^340^ and Ser^356^ in mouse zDHHC2, has been identified in multiple mass spectrometric studies (68–71) (Fig. 2).

In addition to zDHHC2 and zDHHC5, a number of other human zDHHC enzymes possess sequences that are similar to the aforementioned endocytotic signaling motifs. Specifically, zDHHC8 contains C-terminal ^478^SYDSLL and ^541^YDNL sequences, with mass spectrometric evidence of phosphorylation within the vicinity of these sites, including at Tyr^541^ of the YDNL sequence (72–76). zDHHC5, zDHHC14, zDHHC15, and zDHHC20 all contain C-terminal S*XXX*LL sequences (^469^SYDSLL, ^446^SPPRLL, ^303^SQNPLL, and ^330^SKNRLL, respectively), with reported phosphorylation at Ser^446^ in zDHHC14 and Ser^330^ of zDHHC20 (58, 70, 71, 77, 78) (Fig. 2). Therefore, it is possible that regulation of *S*-acyltransferase enzyme localization though C-terminal chain phosphorylation is not only limited to zDHHC2 and zDHHC5 but could in fact be a more widespread phenomenon among the zDHHC enzyme family. However, as in previous cases, validation of many of these reported phosphorylation sites using techniques other than MS is still required.

Analysis of other reported phosphorylation sites in zDHHC enzymes highlights further potential for structural/conformational regulation. The C-terminal Thr-Thr-Xaa-Glu (TT*X*E) motif is a conserved feature of zDHHC enzymes (34) (Fig. 2). Human zDHHC20 enzyme structural data revealed that this motif lies in close proximity to the catalytic DHHC tetrapeptide, where the conformation is stabilized through hydrogen bonding between the second threonine of the TT*X*E motif (Thr^241^) and the aspartate of the catalytic DHHC tetrapeptide (Asp^153^) (39) (Fig. 3*C*). Importantly, alanine substitution of the two threonine amino acids to form an AA*X*E mutant was associated with a marked reduction in enzymatic activity (39). Some zDHHC enzymes possess an analogous Thr-Ser-Xaa-Glu (TS*X*E) motif, where a similar stabilizing hydrogen-bonding interaction between the serine and the DHHC aspartate can be predicted (Fig. 3*D*). Interestingly, there is proteomics evidence to support the phosphorylation of Ser^552^ in the TS*X*E motif of human zDHHC13 (Fig. 2). It is possible that this modification could alter the conformation of the enzyme by disrupting the interaction between the TS*X*E and DHHC motifs, thereby affecting the enzyme's catalytic activity (as seen with the AA*X*E mutant of zDHHC20). This hypothesis was strengthened by modeling a phosphothreonine PTM into the zDHHC20 crystal structure TT*X*E motif. This model shows that hydrogen bonding between Asp^153^ and the phosphorylated form of Thr^241^ appears to be precluded (Fig. 3*E*). Thus, phosphorylation of the TS*X*E motif in zDHHC13 might be a novel mechanism of enzyme regulation, although further experiments are required to validate this phosphorylation site and the possible effects of this modification on zDHHC13 activity.

It is clear that phosphorylation of zDHHC enzymes can be associated with a broad range of regulatory effects. This section has focused on reported effects of phosphorylation on substrate interactions of zDHHC13 and zDHHC3 and dynamic trafficking of zDHHC2 and zDHHC5 and highlighted where these effects might extend to related enzymes. In addition, we provided a more speculative example of potential phosphoregulation of the TS*X*E motif in zDHHC13 and its effects on intramolecular interactions with the DHHC domain of this enzyme. However, these discussions have covered only a fraction of the total residues and zDHHC enzymes that have so far been reported as modified by this PTM type. The effect of the majority of these phosphorylation modifications on zDHHC enzymes is still unknown, and this represents a major knowledge gap, highlighting the need for further research in the area.

## Regulation of zDHHC enzymes by *S*-acylation

**Figure 4.**
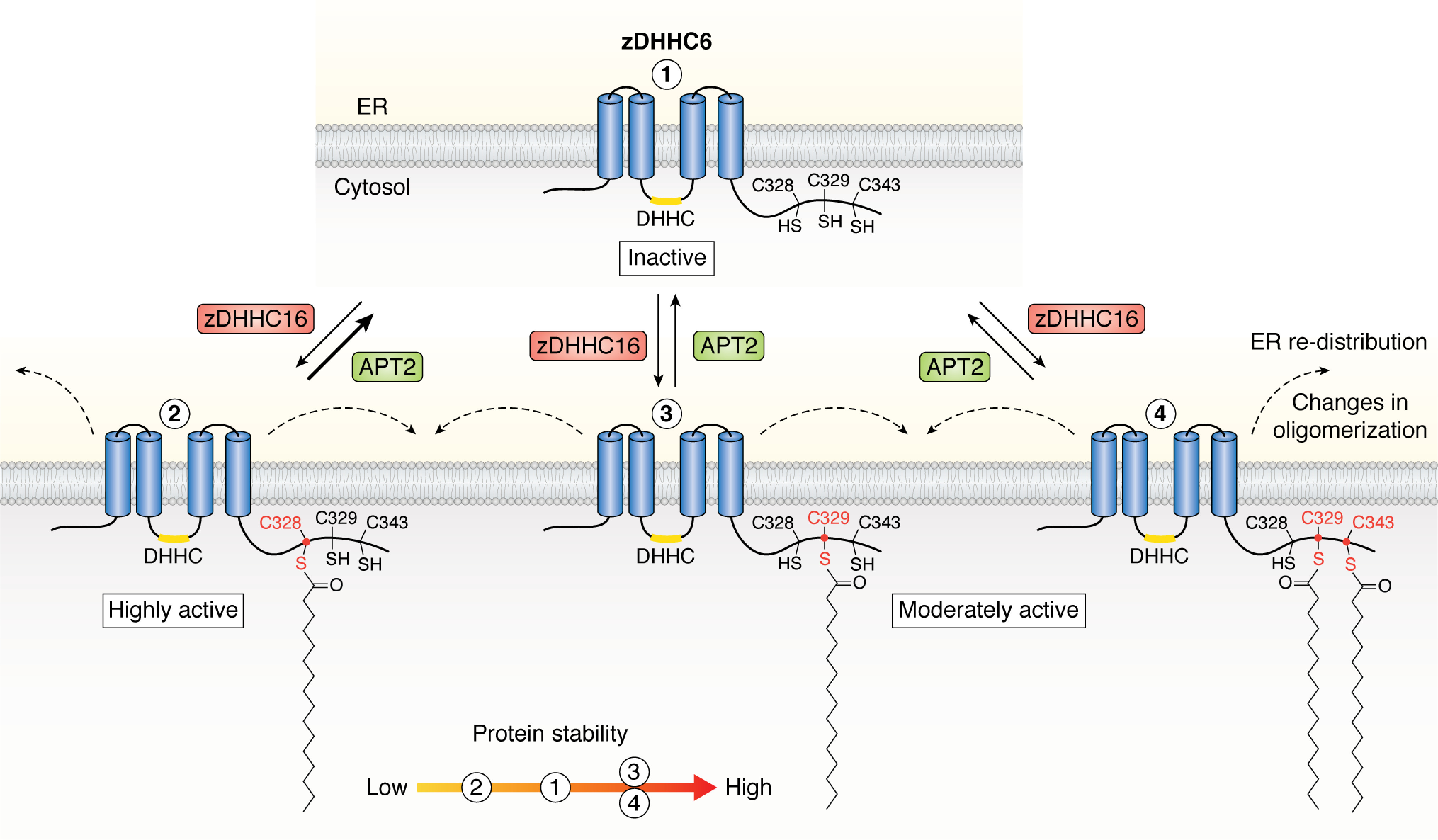
**Representation of the three main forms of *S*-acylated zDHHC6, and the effects of these modifications.** zDHHC6 is localized to endoplasmic reticulum membranes (*ER*), where the nonacylated enzyme (*1*) is inactive and exhibits moderate protein stability. *S*-Acylation of zDHHC6 on the single C-terminal Cys^328^ by zDHHC16 affords a highly active enzyme (*2*) that undergoes both rapid protein degradation and APT2-mediated deacylation. *S*-Acylation of zDHHC6 at Cys^329^ alone (*3*) or Cys^329^ and Cys^343^ (*4*) affords a moderately active and highly stable enzyme. *S*-Acylation of zDHHC6 also results in changes to the oligomeric state and ER distribution of the enzyme (depicted by *dashed arrows*).

Several zDHHC enzymes have been reported to undergo *S*-acylation outside of their catalytic DHHC cysteine. The role of this *S*-acylation as a regulatory modification has been most extensively studied for zDHHC6 using a combination of kinetic measurements of protein and *S*-acylation *t*_½_ of WT and cysteine mutant zDHHC6 combined with mathematical modeling. zDHHC6 is *S*-acylated by zDHHC16 on three cysteine residues that lie within the soluble cytosolic C-terminal tail of the enzyme, namely Cys^328^, Cys^329^, and Cys^343^ (79, 80) (Fig. 2). Interestingly, *S*-acylation of each cysteine turns over with different kinetics, and these modifications differentially affect catalytic activity and protein stability (Fig. 4). Specifically, single *S*-acylation of cysteine Cys^328^ of zDHHC6 leads to a substantial reduction in protein *t*_½_ (5 h *versus* 40 h for nonacylated protein) that is pronounced further when the other two cysteines are also modified (protein *t*_½_ of 0.3 h) (81). zDHHC6 *S*-acylated at Cys^328^ is rapidly degraded by the ubiquitin-dependent endoplasmic reticulum–associated degradation (ERAD) pathway or converted to a different species by acyl-protein thioesterase 2 (APT2)-mediated deacylation (81). In contrast, single *S*-acylation at Cys^329^ enhances zDHHC6 stability by 2.5-fold, whereas single modification of Cys^343^ leads to around a 2-fold reduction in *t*_½_ (81). Intriguingly, although Cys^328^-modified zDHHC6 displays rapid *S*-acylation and protein turnover kinetics, modification of this site generates a highly active form of the enzyme (81). The exact mechanism behind these site-specific modulating effects is unknown, but it is worth noting that a number of proteomics studies have detected zDHHC6 ubiquitination in the C-terminal domain at Lys^245^, Lys^273^, Lys^336^, Lys^353^, and Lys^371^ (82–84) (Fig. 2). Furthermore, zDHHC6 displayed enhanced ubiquitination when the thioesterase APT2 was depleted, highlighting a link between *S*-acylation and ubiquitination of the *S*-acyltransferase (81). The ERAD pathway requires transmembrane proteins to be polyubiquitinated, and it is possible that *S*-acylation of Cys^328^ in the flexible cytosolic C terminus promotes E3 ligase access to the ubiquitination sites. This enhanced ubiquitination could be facilitated by a shift in the distribution of zDHHC6 in the two-dimensional space of the ER membrane or by changes in the oligomeric state of the enzyme, as both of these parameters were shown to be affected in *S*-acylation–null mutants of zDHHC6 (81). The positive correlation between *S*-acylation of Cys^328^ and enhanced degradation of zDHHC6 is interesting, as many proteins display higher ubiquitination and degradation when their *S*-acylation is inhibited (9).

In addition to the three *S*-acylated cysteines in the C-tail of zDHHC6, analysis of site-specific *S*-acylation in mouse forebrain identified C-terminal *S*-acylation sites in an additional seven enzymes (79). Interestingly, five of these enzymes (zDHHC-5, -8, -16, -17, and -20) are modified in a conserved GC*XX*N motif (79, 80). Several recent studies have shown that the C-terminal *S*-acylated cysteines in zDHHC5 (Fig. 2) are important in regulating the distribution of this enzyme between the plasma membrane and internal membranes in nonneuronal cells (85–87). Indeed, one study reported that *S*-acylation of these cysteines occurred in response to isoproterenol stimulation, leading to stabilization of zDHHC5 at the plasma membrane (85). As the C-tail of zDHHC5 is also *S*-acylated in brain, it will be interesting in future work to investigate the interplay between *S*-acylation and phosphorylation of the YDNL endocytic motif in regulating activity-dependent trafficking of this enzyme in neurons.

In addition to regulating the stability and localization of zDHHC enzymes, might *S*-acylation also directly affect *S*-acyltransferase activity? Within the zDHHC enzyme CRD, six highly conserved cysteines together with two histidine residues form the two zinc-finger domains that are involved in coordinating Zn^2+^. These zinc-finger domains conform to a Cys-Cys-His-Cys (CCHC) structure, each interacting with a Zn^2+^ ion in a tetrahedral coordination geometry (39, 88, 89). Based on experimental and computational data acquired from zinc finger–bearing proteins with a similar CCHH arrangement (90, 91), it is presumed that Zn^2+^ binding contributes to the correct folding of zDHHC enzymes during their biosynthesis. In addition to ensuring correct protein folding, these Zn^2+^ ions are also believed to be responsible for maintaining the structural stability of *S*-acyltransferase enzymes and for the favorable positioning of the catalytic DHHC cysteine toward the hydrophobic tepee-like pocket formed by the transmembrane domains (TMDs), where acyl-CoA engagement takes place (39). Moreover, such zinc-binding domains are also known to act as protein recognition motifs, and it is therefore possible that they also play some role in substrate binding.

A study by the Linder group employed an MS approach to detect *S*-acylation of mouse zDHHC3 expressed and purified from insect cells. Almost complete coverage of peptides from the CRD was achieved using this approach, and *S*-acylation was reproducibly detected on Cys^146^ (89) (Fig. 2). Furthermore, mutation of this residue caused a marked loss of *S*-acylation of zDHHC3 (89). Intriguingly, Cys^146^ is predicted to be involved in Zn^2+^ coordination based on structural and sequence data of the human zDHHC20 enzyme. The importance of Cys^146^ and other cysteines linked to Zn^2+^ coordination in zDHHC3 was revealed through their individual substitution with serine residues, which perturbed the structural integrity of the enzyme and inhibited *S*-acyltransferase activity (89). The authors of this study noted the caveat that as Zn^2+^ binding is coupled to correct folding of zDHHC enzymes, any misfolding (*e.g.* due to high-level overexpression) could allow *S*-acylation of cysteines that are normally unavailable for such modification (89). Nevertheless, *S*-acylation has also been detected at Cys^161^ of zDHHC9 in mouse forebrain (79) (Fig. 2), which is also linked to Zn^2+^ coordination and present within a different CCHC zinc finger to Cys^146^ of zDHHC3.

In summary, there is convincing evidence from research on zDHHC5 and zDHHC6 that *S*-acylation of C-terminal cysteines can impact the stability, localization, and/or activity of zDHHC enzymes. Furthermore, as several zDHHC enzymes contain conserved *S*-acylation sites, the effects of this PTM may extend to other members of the zDHHC family. Research showing that cysteines involved in zinc coordination can also be targets for *S*-acylation suggests potentially intriguing mechanisms whereby this modification could directly affect enzyme structural integrity and activity. However, further work is required to determine whether *S*-acylation of these zinc-coordinating cysteines is physiologically relevant.

## Regulation of zDHHC enzymes by ubiquitination

Despite being one of the more prominent PTMs exhibited by the zDHHC enzyme family, the literature investigating the effects of ubiquitination on *S*-acyltransferases is limited. As discussed above, there is evidence that the extent of *S*-acylation of zDHHC6 could affect ubiquitination of specific lysines and subsequent enzyme degradation via ERAD (81). In this case, it will be interesting to explore how arginine substitutions of some of these lysines affect the *t*_½_ of zDHHC6 WT and cysteine mutant proteins.

The zDHHC9 enzyme can form a complex with the accessory protein GCP16, which was shown to be important in stabilizing the *S*-acyltransferase and preventing its degradation (92). This effect may be linked to ubiquitination, as a study using the analogous yeast (*S. cerevisiae*) protein complex pair Erf2-Erf4 showed a similar stabilizing effect, with the *t*_½_ of Erf2 decreasing by approximately 6-fold in an Erf4 mutant strain (93). Using His_6_-tagged ubiquitin, it was shown that Erf2 was polyubiquitinated in the Erf4 mutant (93). Ubiquitination was blocked when six lysine residues within the 59-amino acid C terminus of Erf2 (Lys^304^, Lys^311^, Lys^316^, Lys^335^, Lys^355^, and Lys^358^) were mutated to arginines (93). Furthermore, disruption of components of the ERAD system in the Erf4 mutant strain led to an increase in Erf2 *t*_½_, suggesting that ubiquitination targets Erf2 for destruction via the ERAD pathway (93). It was proposed that Erf4 imparts stability on Erf2 by masking these ubiquitination sites, either directly through binding to the C-terminal domain or indirectly by binding to other regions of the protein and imposing conformational changes in the terminal chain (93). The observed stabilizing effects of GCP16 on zDHHC9 may also reflect interplay with the ubiquitination machinery. Indeed, mass spectrometric screens of two human cell lines identified C-terminal ubiquitination of zDHHC9 at Lys^267^ and Lys^281^ (82) (Fig. 2), the latter of which is homologous to the putative Erf2 Lys^316^ ubiquitination site. It will be interesting to explore whether modification of these sites in zDHHC9 is affected by GCP16 depletion.

Interestingly, the ubiquitinated Lys^281^ of zDHHC9 lies within the palmitoyl conserved C terminus (PaCCT) motif that has been shown to be important for *S*-acyltransferase catalytic activity (39, 94). The corresponding lysine in zDHHC20 (Lys^265^) has also been reported to undergo ubiquitination (84), and the crystal structure of zDHHC20 revealed that this lysine forms part of the enzyme's amphipathic α′2 helix. This helix makes contacts with TMD3 and TMD4, which play an important role in accommodating the acyl chain within the transmembrane tepee cavity and also contribute to acyl chain specificity (24, 39). The α′2 helix is stabilized via hydrogen bonding between Asn^266^ and Ser^260^ and Leu^261^, and disruption of the helix by mutation of Asn^266^ to alanine was shown to reduce activity of zDHHC20 (39). Therefore, in addition to the well-established role of ubiquitination in mediating protein degradation, it is plausible that the addition of a bulky 8.6-kDa ubiquitin to zDHHC9 Lys^281^ and zDHHC20 Lys^265^ residues could also perturb catalytic activity through disruption of the α′2 helix structure.

Lysine ubiquitination has also been reported in the vicinity of other critical domains of human *S*-acyltransferases, such as the zDHHC2 and zDHHC20 S*XXX*LL motif (82, 95, 96); the zDHHC5, zDHHC6, zDHHC7, and zDHHC8 TT*X*E motif (58, 82–84, 95–99); and the zDHHC7, zDHHC16, and zDHHC20 zinc-binding CRD (84) (Fig. 2). Again, it is possible that conformational changes of these other motifs induced by ubiquitination may have an effect on the trafficking and activity of these enzymes or that ubiquitination could impart direct effects on enzyme localization. Conversely, changes in protein interactions at these sites or conformational changes (*e.g.* linked to misfolding) could expose lysine residues for ubiquitinatin.

In summary, analysis of Erf2-Erf4 has clearly shown an important role for interplay between accessory protein binding and ubiquitination-mediated zDHHC enzyme degradation, and by analogy this may also operate for zDHHC9/GCP16. In addition, interplay between ubiquitination and *S*-acylation is central to stability of the zDHHC6 enzyme. The detection of ubiquitination on other zDHHC enzymes suggests a wider role for this modification in controlling zDHHC degradation. In addition, as ubiquitination often occurs in regions of zDHHC enzymes linked to activity and trafficking, this PTM could also impact other aspects of zDHHC biology either by disrupting the integrity of important sequences or motifs or by directly affecting enzyme localization. Further research is required to explore these potential regulatory effects.

## Regulation of zDHHC enzymes by methylation and acetylation

A number of unpublished (CST curation sets 2477, 2441, 18334, 2378, 3507, 4590, 10351, 5209, 8175, 18851, 20095, 4999, and 5153; PhosphoSitePlus; RRID:SCR_001837) and published (55, 100–104) mass spectrometric experiments have identified several methylation and acetylation modifications of zDHHC enzymes. Although little is known about the role of these modifications, their potential for zDHHC enzyme regulation will be discussed briefly.

A peptide array methylation experiment revealed that zDHHC8 is a substrate for the SET7/9 (KMT7) protein lysine methyltransferase in humans (105). By mutating putative lysine methylation sites to arginines, Lys^300^ was identified as the residue in zDHHC8 that was modified by SET7/9. Although this methylation site does not occur in established regulatory domains of zDHHC8 (Fig. 2), it will be of interest to determine the effects of this modification on the localization, stability, and activity of this enzyme.

Lysine acetylation can stabilize proteins by either directly competing with ubiquitination or inducing conformational changes that obstruct access to ubiquitin E3 ligases (50). Interestingly, human zDHHC3 Lys^121^ and zDHHC5 Lys^97^ have been shown to be both acetylation and ubiquitination sites in separate proteomics studies (58, 83, 84, 95, 97, 106) (CST curation set 5209) (Fig. 2), which hints toward possible competitive regulation of stability of these enzymes. Paradoxically, lysine acetylation has also been reported to drive protein degradation by aiding the recruitment of third-party proteins that can in turn facilitate ubiquitination (50). Indeed, zDHHC3 and zDHHC5 can also be acetylated (106, 107) (CST curation sets 10351 and 5209) and ubiquitinated (58, 82–84, 95–99) at a number of independent sites, and it is plausible that lysine acetylation could play a role in driving ubiquitination of these enzymes by protein recruitment. It is worth noting that methylation has also been implicated in either stabilizing proteins or promoting their degradation, and such effects should not be disregarded in methylated zDHHC enzymes. Moreover, methylation and acetylation of amino acids can alter their properties in a manner that makes new interactions possible (54, 55), and it is plausible that this could facilitate binding of protein substrates to the *S*-acyltransferases.

In summary, although zDHHC8 lysine methylation has been confirmed experimentally and the modifying enzyme has been identified, the functional relevance of acetylation and methylation of any zDHHC enzyme is not yet known (Fig. 1*B*). However, given that several zDHHC enzymes are modified (Fig. 1*A*), exploring the effects of methylation and acetylation is expected to reveal new insight into the regulatory effects of these PTMs on zDHHC enzymes.

## Perspective

Although our understanding of how zDHHC enzymes are regulated has lagged behind our appreciation of the substrate targets and cellular functions of zDHHC enzymes, there is emerging evidence showing important regulatory effects of PTMs (Fig. 1*B*). These PTMs affect many aspects of zDHHC enzyme biology, including trafficking, localization, substrate binding, activity, and stability/degradation. However, this is likely to represent only the tip of the iceberg. The available PTM proteome data sets combined with improved understanding of the structure-function relationship of zDHHC enzymes provides a wealth of information that can help generate a much better understanding of regulatory mechanisms controlling this important class of enzymes.

## Data availability

All data are contained within the article.
